# A Case of Severe Organ Dysfunction and Skin Lesions Due to Methotrexate Toxicity

**DOI:** 10.7759/cureus.60008

**Published:** 2024-05-09

**Authors:** Tullio R C. Barros, Yasmin P Ribeiro, Vilson C Oliveira, Marcela A. Lopes

**Affiliations:** 1 Internal Medicine, Hospital e Maternidade Salvalus, São Paulo, BRA; 2 Medicine, Universidade de São Paulo, São Paulo, BRA; 3 Geriatrics, Hospital e Maternidade Salvalus, São Paulo, BRA; 4 Critical Care, Hospital e Maternidade Salvalus, São Paulo, BRA

**Keywords:** hepatotoxicity, pancytopenia, mucositis, psoriasis, methotrexate intoxication

## Abstract

Methotrexate is an anti-inflammatory and immunomodulatory drug, widely used for moderate to severe psoriasis and other rheumatological conditions such as rheumatoid arthritis, besides some types of malignancies. Side effects are more prevalent in high acute doses but can also be seen in low-dose chronic use, especially in cases of drug-dosing errors. Possible symptoms of toxicity include gastrointestinal, hepatic, hematologic and renal dysfunctions, but may also include mucositis and worsening of the psoriatic lesions. Here, we describe a case involving methotrexate toxicity in an elderly patient with psoriasis, detailing the management.

## Introduction

Psoriasis is a chronic skin disease that affects up to 1.3% of the Brazilian population [1]. It manifests as erythematous plaques with overlying silver scales, preferentially involving joints, scalp and genitals. It is associated with other comorbidities, such as arthritis, due to its systemic inflammatory etiology. The immune-mediated activation of T-cells and the release of multiple cytokines promote hyperproliferation of epidermal keratinocytes [2].

Methotrexate (MTX) is an anti-folate drug with anti-inflammatory and immunomodulatory properties. Although its pharmacological mechanisms are not completely understood, some explanations have been proposed. Once it enters the cell via folate carriers, MTX inhibits the conversion of dihydrofolate to tetrahydrofolate, a crucial step in purine biosynthesis. Therefore, it blocks DNA synthesis and repair [3]. Additionally, MTX is thought to promote intracellular adenosine release and this receptor has anti-inflammatory actions [4].

Due to its inhibition of rapidly proliferating cells and direct action on T-lymphocytes, MTX has been widely prescribed by rheumatologists to treat various rheumatic conditions, such as psoriasis, since 1951 [3]. It may also improve the efficacy of other drugs used concomitantly for those diseases. In their treatment, it is administered as a long-term low-dose therapy (usually 7.5-25 mg/week), but it is also used for leukemia, lymphomas, and other malignancies in cyclic, higher doses (usually greater than 500 mg/m²) [5,6]. In psoriasis, it is usually prescribed for moderate to severe cases, where more than 5% to 10% of the body surface is affected or in the case of disabling disease. There are numerous topical therapies, as well as systemic ones, available, which include MTX, retinoids, ciclosporin, fumaric acid esters, and biologic agents [7].

Side effects of MTX are not usually observed in low doses (up to 25 mg/week), but are more prevalent in high doses (above 500 mg/m²). However, even at the same doses, patients may exhibit different responses and patterns of toxicity, due to polymorphism in the genes involved in drug metabolism [8]. In a 2006 review, 50% of individuals who used methotrexate reported some side effects, with 34% of them being significant [9,10]. In another case report, in India, seven intoxicated patients who suffered from psoriasis were studied and the cumulative dose that led to acute toxicity was 35-105 mg taken over three to seven days [5]. Here, we present a case of methotrexate toxicity, from São Paulo, Brazil.

## Case presentation

A 69-year-old man was admitted with complaints of multiple painful skin lesions persisting for the past month. These lesions predominantly affected the mouth, genitalia, and limbs, and were accompanied by symptoms of fatigue and reduced appetite, leading to a decreased fluid intake. His medical history included hypertension, hypercholesterolemia, and a recent diagnosis of psoriasis, for which he had been prescribed methotrexate. However, it was discovered that he had been inadvertently taking an incorrect dosage of the medication since the start of the treatment, due to a misunderstanding of the dosage regimen in the prescription. He had been self-administering a dosage of 2.5 mg, three pills in the morning and three in the evening daily, totaling 105 mg per week for a month, significantly exceeding the typical weekly dose range of 7.5 to 25 mg.

**Figure 1:**
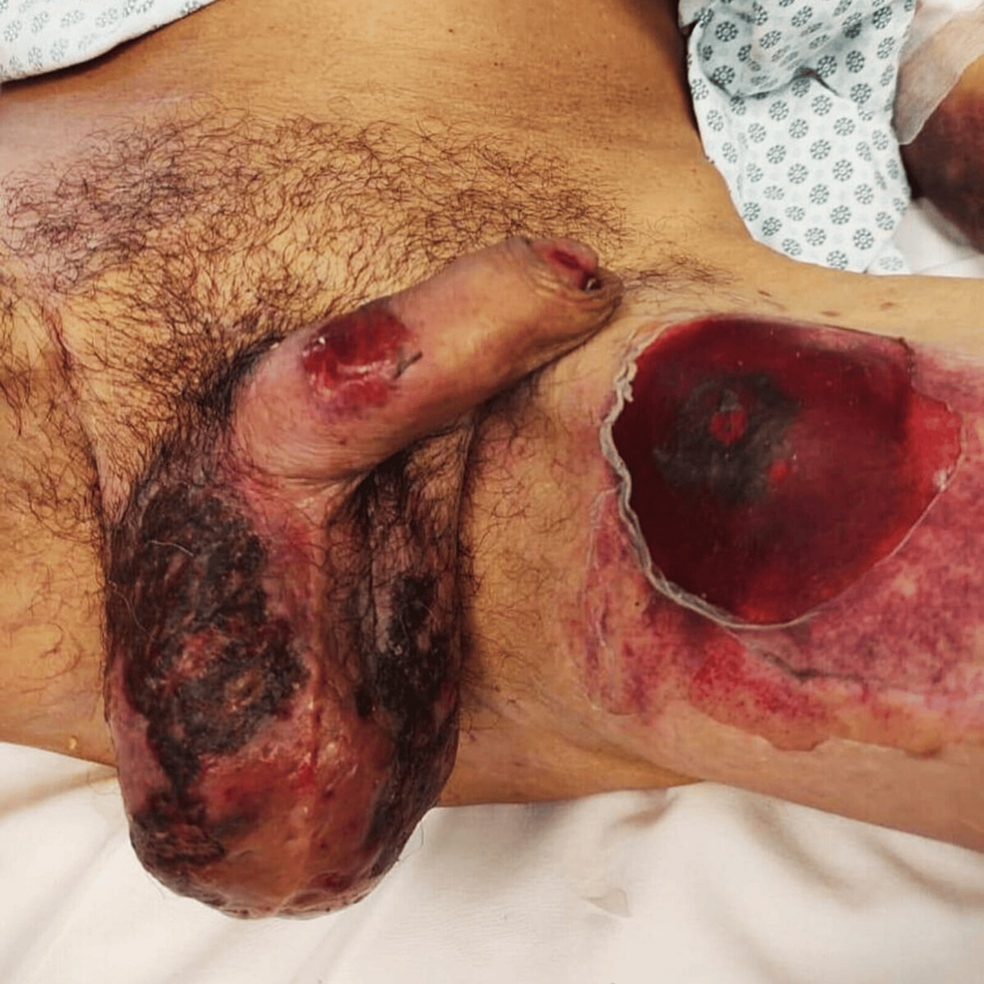
Blistering and scaly lesions on the perineal region and left inner thigh

**Figure 2:**
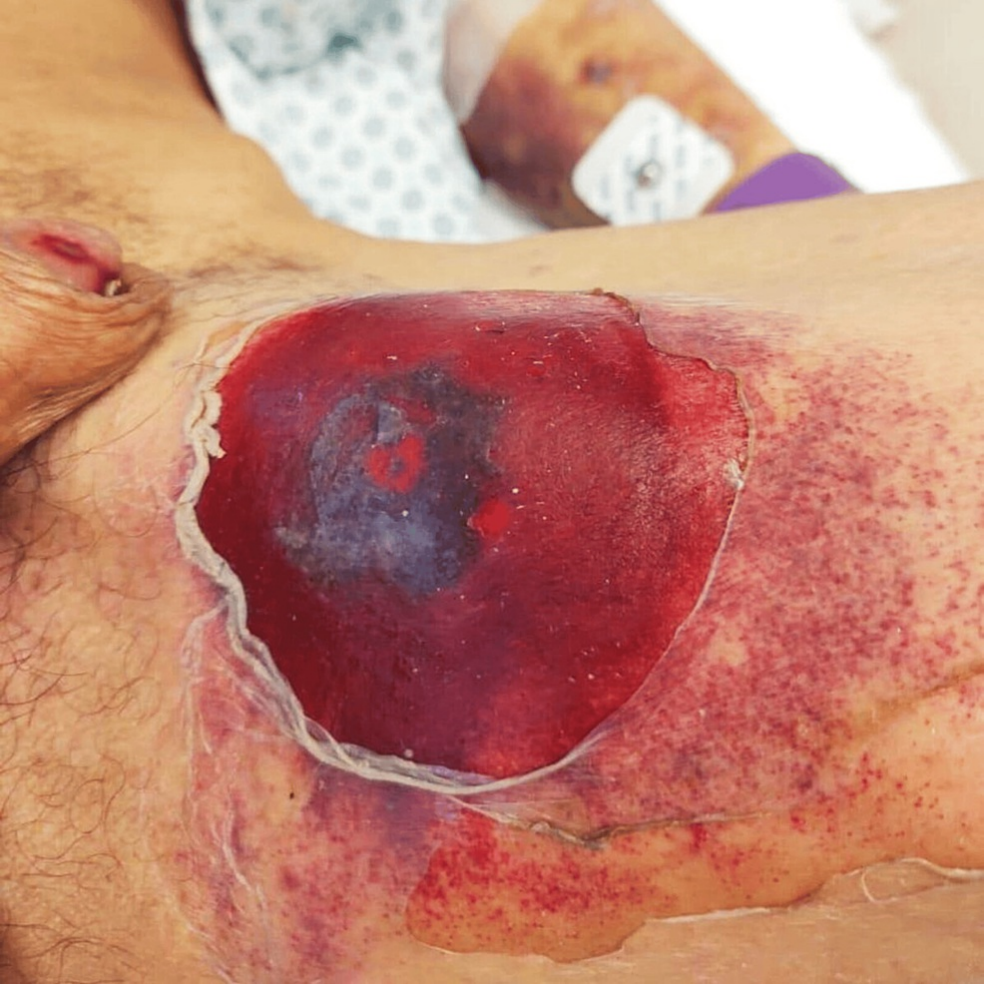
Blister and erythematous scaly lesion on the left inner thigh

Upon clinical examination, the patient exhibited a wide variety of severe skin lesions, notably several blistering and erythematous scaly lesions on various body parts, including the mouth, arms, legs, and genital region (Figures 1, 2).

**Figure 3:**
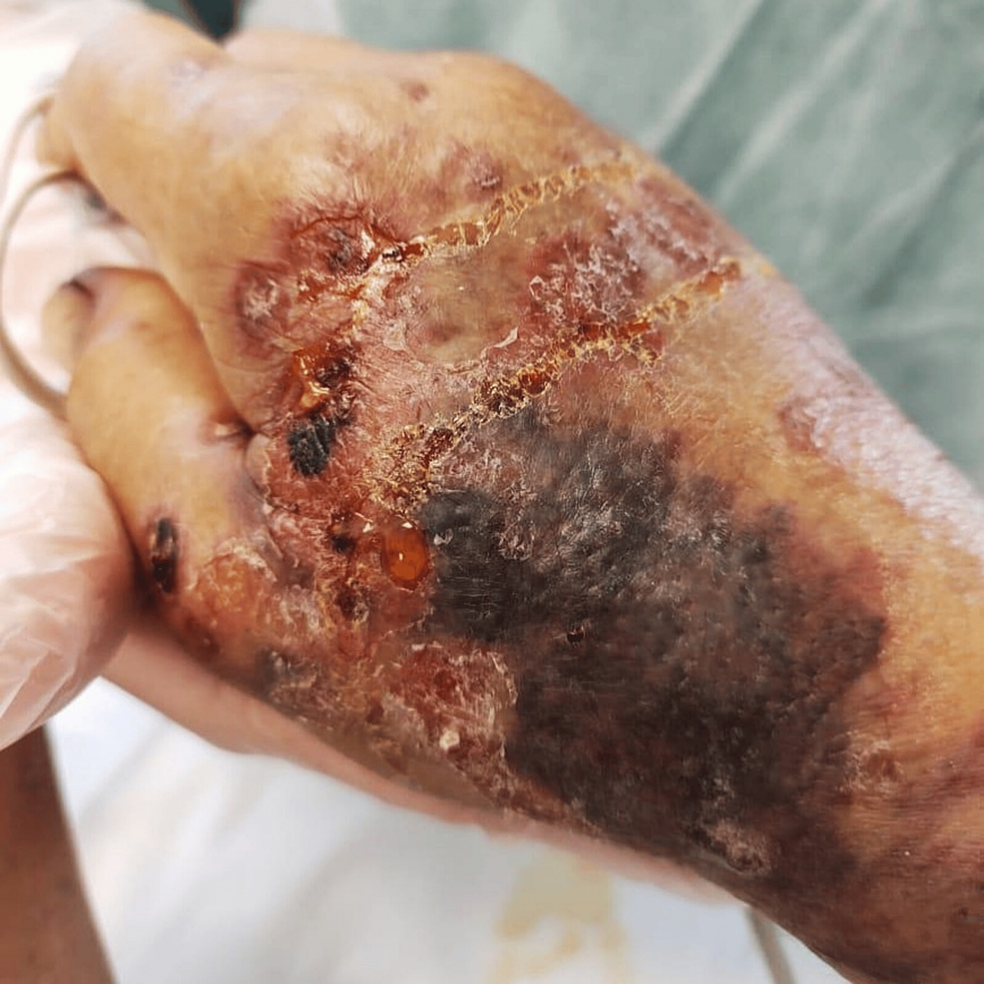
Blisters and ulcerations over psoriatic lesions on the right fist

**Figure 4:**
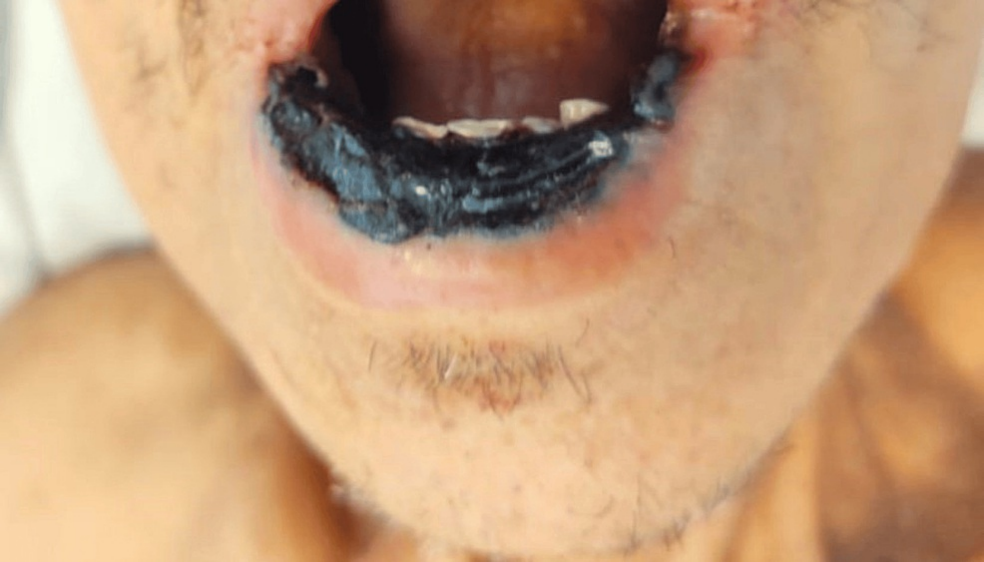
Severe mucositis with necrotic lesions on the lips

**Figure 5:**
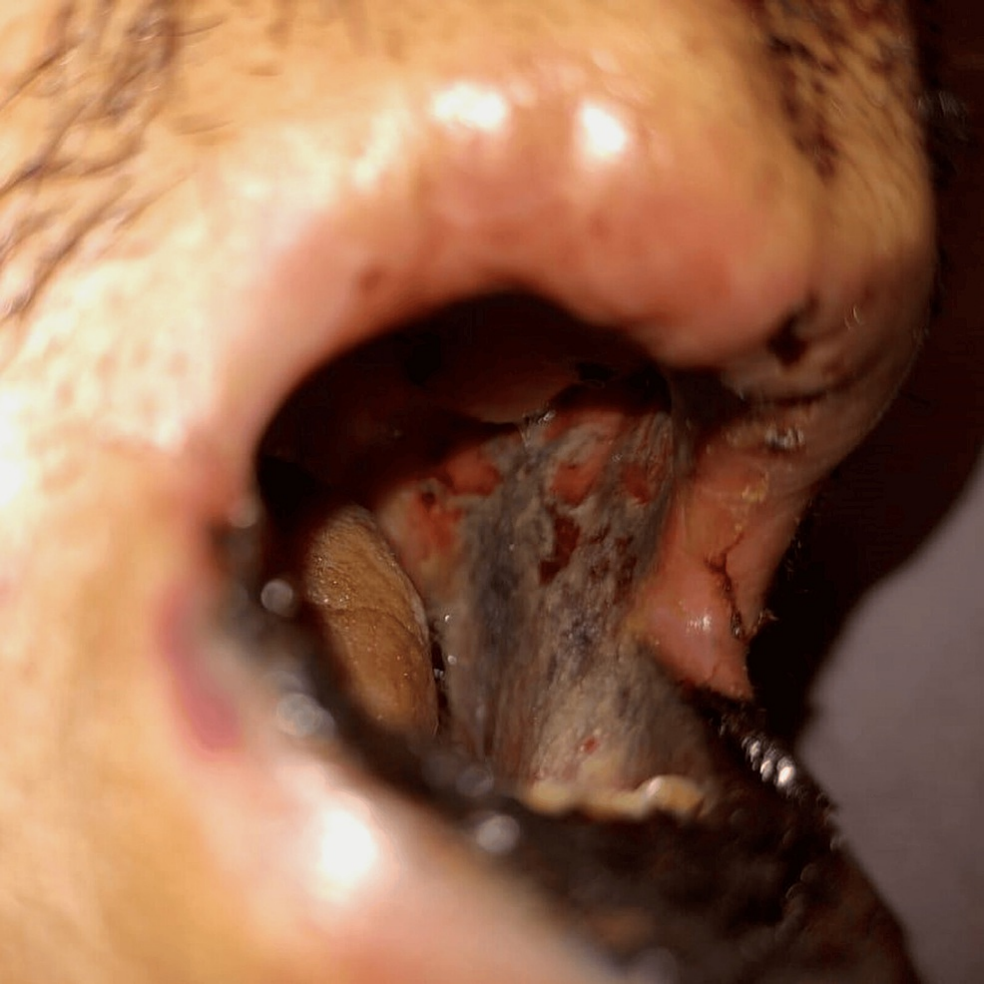
Extensive oral mucositis

Some of those lesions superimposed psoriatic plaques on extensor areas, like on the fists, resulting in hematic blisters and scaly lesions with ulcerated necrotic regions (Figure 3). The mucosal involvement was also extensive: severe mucositis affected the entire oral cavity with necrotic deterioration of the lips (Figures 4, 5). Consequently, evident sings of dehydration were also observed.

Laboratory tests were promptly done, revealing intense myelosuppression, as evidenced by leukopenia (100/mm³), and thrombocytopenia (3000/mm³). Other findings were liver dysfunction (international normalized ratio, or INR, 1.58; alanine aminotransferase, or ALT, 54 U/L; total bilirubin, 3.9 mg/dL), and acute kidney injury (creatinine, 1.5 mg/dL) due to the probable pre-renal mechanism by dehydration.

After this initial investigation, the diagnosis was clearly defined as MTX toxicity, which was compatible with the excessive dose that our patient had been using for so long and it explained all the clinical and laboratory findings (skin lesions, mucositis, pancytopenia and liver failure). We started therapeutically folic acid, laser therapy for oral lesions, and topical care for skin lesions, in addition to intravenous hydration and intensive organ support.

## Discussion

The initial MTX treatment for psoriasis usually starts with 7.5 mg weekly, ranging to 15 mg, and is adjusted weekly to monthly, if necessary, reaching the maximum dose of 25 mg per week [10]. It can be administered once a week, which is preconized, or divided in daily doses (although proven less effective) [2]. The most common route of administration is oral, but some guidelines prefer subcutaneous injections due to the risk of pill overdosing by the patient and the advantage of reducing gastrointestinal effects [2,11]. Because long-time intracellular storage of MTX metabolites leads to chronic folate depletion, folic acid supplementation is advised along with the treatment (at least 5 mg per week) [6,8].

As in our case, the most common reason for MTX toxicity seems to be poor patient counselling regarding the medication (doses, schedules, and potential collateral effects) and what to expect from the disease, alongside accidental overdose [5,10]. Apart from dosing errors, renal insufficiency, advanced age, folate deficiency, hypovolemia and drug-interactions are some of the other risk factors [12].

Hepatic side effects were identified in psoriasis patients in 1971. Long-term low doses cause more hepatotoxicity than high doses and it is the most common serious adverse effect [3]. Clinical manifestations include elevated transaminases, increased bilirubin, as seen in our patient, and even steatosis and fibrosis; in pathology, Ito cells hypertrophy may be found [4]. The mechanisms explaining hepatotoxicity are not known, but it is very common (60% of all patients taking high doses experience reversible hepatitis, 25% experience hyperbilirubinemia and about 70% of patients taking low doses for rheumatoid arthritis experience some kind of liver damage) [3,4,6]. Previous liver disease (infection with hepatitis B and C viruses, alcoholic disease), concomitant use of other anti-rheumatic drugs, obesity, diabetes, and hyperlipidemia are risk factors for developing hepatotoxicity with MTX [3].

Dermatological effects are unusual, especially with low doses [6]. However, there have been several cases reported where patients with psoriasis experienced worsening of the previous skin lesions, with ulceration of the psoriatic plaques [10]. Other manifestations can be rashes, Stevens-Johnson syndrome, etc.; histologically, keratinocyte enlargement and epidermal necrolysis can be observed [6]. In a case series study, patients with psoriasis with an overdose of MTX presented with ulceration and bleeding of the psoriatic plaques, oral ulcers and some even had genital ulcers [5]. Mucocutaneous ulcers can range from oral to intestinal mucositis, affecting any part of the gastrointestinal tract, as seen in our patient with esophageal ulcers [4].

The most serious and fatal risk is usually myelosuppression. Our patient's lab results showed leukopenia and thrombocytopenia, which are the major dose-limiting factors in the therapy, with life-threatening consequences. Myelosuppression is more common in high-dose schemes and is considered rare in low doses. Some risk factors for its occurrence are advanced age, impaired kidney function, concomitant treatment with other anti-rheumatic drugs, current infections, and folic acid deficiency [4].

There are some other possible symptoms of MTX toxicity, not shown by our patient, the most common being gastrointestinal ones (nausea, vomiting, diarrhea, and abdominal discomfort) [4]. Nephrotoxicity is also typical, especially in patients with impaired renal function or with the use of other drugs that reduce renal elimination (NSAIDs and sulfonamides, for example), and it furthers toxicity to other organs with diminished MTX clearance [4,6]. Neurological symptoms like headaches, fever, malaise, even seizures and hemiparesis are rarer and can occur mainly in high-dose schemes [3,10]. Pulmonary effects, the typical being hypersensitivity pneumonitis, are very unusual [13,14].

Regarding treatment, there isn’t a specific threshold mentioned in guidelines for initiating it, particularly in acute toxicities. Management can range from observation in mild cases to more aggressive interventions in severe cases [12]. Severe cases, like our patient’s, should be admitted to an ICU, with the goal of clearing the drug from the bloodstream, replenishing folic acid, and treating organ damages [6]. The antidote of choice in acute MTX poisoning is folic or folinic acid, and there is no difference in their efficacy [2,6]. However, an adequate renal function is necessary for them to fully work [4]. The patient’s reduced creatinine clearance might be a factor interfering with the therapy success.

Mucositis can be extremely serious, leading to infections and even death, and even though many types of treatment have been reviewed, there is no standard practice. Some studies show great benefits of laser therapy in not only treating but also preventing oral lesions in MTX users, as employed for our patient [15,16]. Additional measures, like topical care for skin lesions and further supportive treatments, should be individualized based on patients’ risk factors [3].

## Conclusions

Methotrexate is an affordable drug for treating moderate to severe cases of psoriasis, with its benefits well established. However, MTX toxicity can be highly detrimental to a patient’s health and can be even life-threatening, with hematological, hepatic, and renal dysfunctions, among other important effects. In conclusion, this case provides valuable insights into the potential consequences of high-dose methotrexate and serves as a didactic and self-explanatory lesson, emphasizing the importance of adhering to recommended doses and close monitoring of the patient to prevent adverse outcomes.
